# Comprehensive assessment of unfolded protein response and its association with tumor progression in pan‐cancer

**DOI:** 10.1002/imo2.70084

**Published:** 2026-02-20

**Authors:** Xinyu Yang, Faming Zhao, Jing Yang, Xinran Xia, Liwei Chen, Peng Zeng, Liang Chen, Yanxia Hu, Xia Sheng

**Affiliations:** ^1^ Department of Urology, Tongji Hospital, Tongji Medical College Huazhong University of Science and Technology Wuhan China; ^2^ School of Public Health, Tongji Medical College Huazhong University of Science and Technology Wuhan China; ^3^ Institute of Biomedical Research, School of Life and Health Sciences Hainan University Haikou China

**Keywords:** endoplasmic reticulum stress, machine learning, pan‐cancer, single‐cell transcriptomics, unfolded protein response

## Abstract

Disruption of proteostasis in the endoplasmic reticulum leads to the activation of the unfolded protein response (UPR). UPR activation is common in malignant cells and is often considered a contributor to cancer progression. However, the accurate evaluation of UPR status in pan‐cancer is still lacking. Furthermore, the UPR‐related key characteristics in cancer progression remain to be explored. To fill in these gaps, we first proposed a UPR signature, according to which a UPR score was assigned to each tumor/tumor cell by gene set variation analysis. Based on this, we depicted the UPR landscape across 33 kinds of tumors and 28 kinds of normal tissues, which revealed significant inter‐ and intra‐tumoral heterogeneity in UPR activity across different cancers. Next, by integrating bulk and single‐cell transcriptomic data, we unveiled robust correlations of UPR activation with critical genomic and immune features amongst different cancers. In addition, by employing three machine learning algorithms, we identified and validated a promising biomarker derived from the perspective of UPR for breast cancer prognosis. In summary, our study offers an effective approach for evaluating UPR activity in human cancer and underscores the clinical significance of UPR activity in cancer progression.

## Highlights

- Developed a transcriptome‐based unfolded protein response (UPR) signature that enables reliable assessment of UPR activation across pan‐cancer.
- Systematically characterized the landscape of UPR heterogeneity and uncovered its multidimensional associations with key tumor features.
- Identified and validated a UPR‐related biomarker with significant prognostic value in breast cancer.

## Graphical abstract

Schematic of the study design. In this study, we developed a comprehensive unfolded protein response (UPR) gene signature and applied it to systematically characterize the UPR landscape across multiple cancer types. By integrating bulk and single‐cell transcriptomic data, we revealed substantial inter‐ and intra‐tumoral heterogeneity in UPR activity and uncovered critical associations between UPR and key cancer features, including genomic features and immune responses. Importantly, we further employed machine learning methods to construct a simple three‐gene prognostic model derived from the UPR signature, which can effectively predict overall survival of breast cancer patients, highlighting both the role of UPR in modulating tumor immunity and the clinical utility of the UPR gene signature in cancer prognosis and precision medicine.

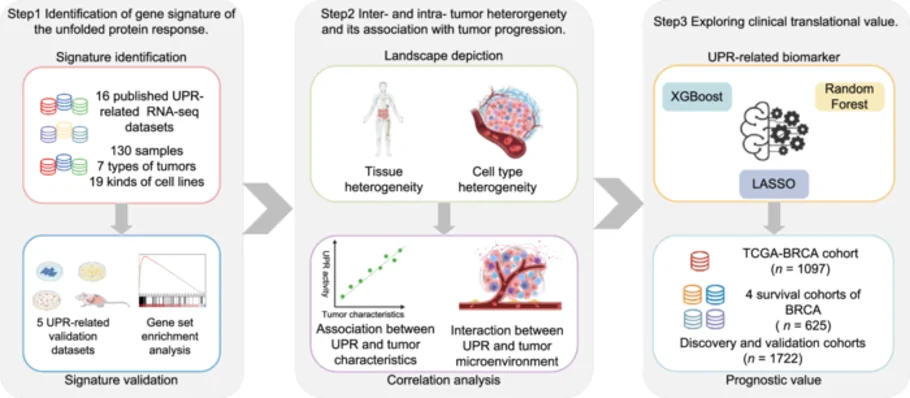

## 1 INTRODUCTION

The endoplasmic reticulum (ER) plays a central role in maintaining proteostasis, calcium metabolism, as well as the synthesis of lipids and glucose [1]. Various external and internal stimuli can disrupt the protein folding capacity of the ER, leading to the accumulation of misfolded and unfolded proteins, thereby triggering ER stress and activating the unfolded protein response (UPR) [2]. Under physiological conditions, transient activation of the UPR serves as a protective mechanism to restore homeostasis by upregulating molecular chaperones, reducing the secretory protein load, and enhancing ER‐associated degradation (ERAD) and autophagy [3]. This adaptive response is particularly critical in secretory tissues such as the liver, pancreas, and prostate, which are under constant proteostatic stress [4]. However, under chronic or unresolved ER stress, prolonged activation of UPR branches, including the PERK‐ATF4 and IRE1‐XBP1 pathways, can shift from a cytoprotective to a pro‐tumorigenic role, promoting survival, metabolic reprogramming, and inflammation that collectively facilitate malignant transformation and tumor progression [5, 6]. Adverse conditions such as glucose or oxygen deprivation, along with calcium imbalance, further exacerbate proteostatic stress in tumor cells, where the UPR can determine cell fate by either restoring homeostasis or inducing apoptosis.

In mammals, there are three sensors for ER stress: inositol‐requiring protein 1α (IRE1α), activating transcription factor 6 (ATF6), and protein kinase R‐like ER kinase (PERK) [7, 8]. Under ER stress, the luminal domain of the ER stress sensors, which are initially bound by the molecular chaperone glucose‐regulated protein 78, becomes dissociated and activated, thus initiating downstream UPR signaling to restore and maintain ER homeostasis [9]. Upon ER stress, the expression levels of UPR downstream transcription factors, such as spliced XBP1 (XBP1s), activating transcription factor 4, or ATF6, can be upregulated or activated, thereby promoting the transcription of target genes involved in protein folding, antioxidant response, as well as amino acid synthesis and lipid metabolism [10]. This progress strengthens the protection against subsequent damage and helps maintain cellular homeostasis. On the other hand, UPR can activate the downstream pro‐apoptotic signaling in response to irremediable stress, ultimately leading to programmed cell death [11].

Accumulating evidence has shown that the UPR plays multiple roles in the progression of human cancers, and targeting the UPR can mitigate tumor progression in experimental and clinical settings [12]. For example, the IRE1α‐XBP1s branch has been shown to promote the progression of breast, prostate, and liver cancers by interacting with key oncogenic pathways, including the HIF1α, c‐MYC, and canonical Wnt pathways [13, 14, 15]. Therefore, therapeutic targeting of this axis in patients with advanced solid tumors is under clinical evaluation [16]. In addition to the functional implication, UPR status also appears to have prognostic indications. For instance, our previous studies have shown that UPR scores hold high prognostic value in head and neck cancer and osteosarcoma, where patients with higher UPR scores often exhibit more advanced clinical stages and poorer prognoses [17, 18]. However, only limited studies have systematically investigated the role of UPR on the pan‐cancer scale [19, 20]. For instance, an analysis of the Cancer Genome Atlas (TCGA) pan‐cancer dataset identified UPR as a mechanistic link between tumor aneuploidy and immune dysregulation, where the PERK pathway was proposed as a key driver of immunosuppression [20]. Therefore, a comprehensive pan‐cancer, multi‐omics characterization of UPR activation across diverse tumor types remains largely unexplored.

It is generally acknowledged that sustained and moderate ER stress facilitates cancer progression via several mechanisms, such as cell proliferation, metastasis, therapy resistance, angiogenesis, and immune evasion [21]. This makes it crucial to systematically evaluate the association between UPR and tumor progression and explore its clinical significance. However, most of the previous UPR gene signatures used to evaluate UPR activity were derived from bulk RNA sequencing (RNA‐seq) data with limited generalizability [22]. Identifying a comprehensive UPR signature in pan‐cancer and investigating its clinical significance remains an important and unresolved issue. Additionally, previous studies on the relationship between UPR and tumor characteristics focus on one type of tumor [23, 24], and there currently lacks a comprehensive transcriptomic analysis to elaborate on UPR‐related molecular alterations in cancer yet.

To fill in these gaps, the present study integrated 16 published UPR‐related RNA‐seq datasets, comprising 130 samples from 7 types of tumors and 19 cell lines. Then, we identified 159 genes associated with UPR activation status to construct a gene signature and validated its effectiveness through external validation datasets. Subsequently, by integrating bulk RNA‐seq data, scRNA‐seq, and multiple algorithms, we systematically depicted the UPR landscape and revealed the inter‐ and intra‐tumoral heterogeneity of UPR activity. Furthermore, we revealed tumor‐specific correlations of UPR levels with both oncogenic pathways and immune features. Lastly, we utilized machine learning algorithms to uncover the prognostic potential of hub genes within the UPR signature in tumors. Overall, by developing a UPR gene signature, this study not only systematically characterized the UPR landscape across cancers but also uncovered its key associations with major cancer‐related features.

## 2 RESULTS

### 2.1 Identification of a gene signature to estimate UPR status in cancer

To date, most of the gene signatures developed to evaluate UPR activation were derived from RNA‐seq data of a single tumor type, with relatively limited generalizability. To overcome this, we re‐processed and integrated high‐throughput transcriptomics datasets from 16 different studies comprising 130 RNA‐seq samples. These datasets included 19 types of cancer cell lines exposed to typical UPR inducers, such as thapsigargin (TG), tunicamycin (TM), hypoxia, and glucose deprivation (GD) (Table S1). The principal component analysis uncovered a notable batch effect of data from different sources, obscuring the biological difference (Figure S1A). Notably, after removing the batch effect by the “ComBat” function of the R package “sva,” the influence of sources was mitigated, and the difference in biological states emerged (Figure S1B). In the procedure of WGCNA, a soft threshold β of 12 was chosen, which yielded an appropriate power value for the construction of the co‐expression network (Figure S2A). Furthermore, we computed the correlations of each module with both the cell status and the UPR activity that was calculated based on published gene sets. The blue module showed the highest correlation with the UPR activation (Figure 1A, Figure S2B,C). To further evaluate robustness, we excluded central nervous system tumor cell lines and repeated WGCNA in a sensitivity analysis. The resulting blue module remained strongly associated with UPR activation and largely recapitulated the original module, differing by only eight genes (Figure S2D,E). Within the blue module, the correlation coefficient between gene significance (GS) and module membership (MM) was 0.56, indicating that the module was constructed with relatively high quality (Figure 1B). Based on this, we selected 159 genes strongly associated with UPR activation as the UPR signature, characterized by GS > 0.4 and MM > 0.7 (Figure 1B and Table S2).

**FIGURE 1:**
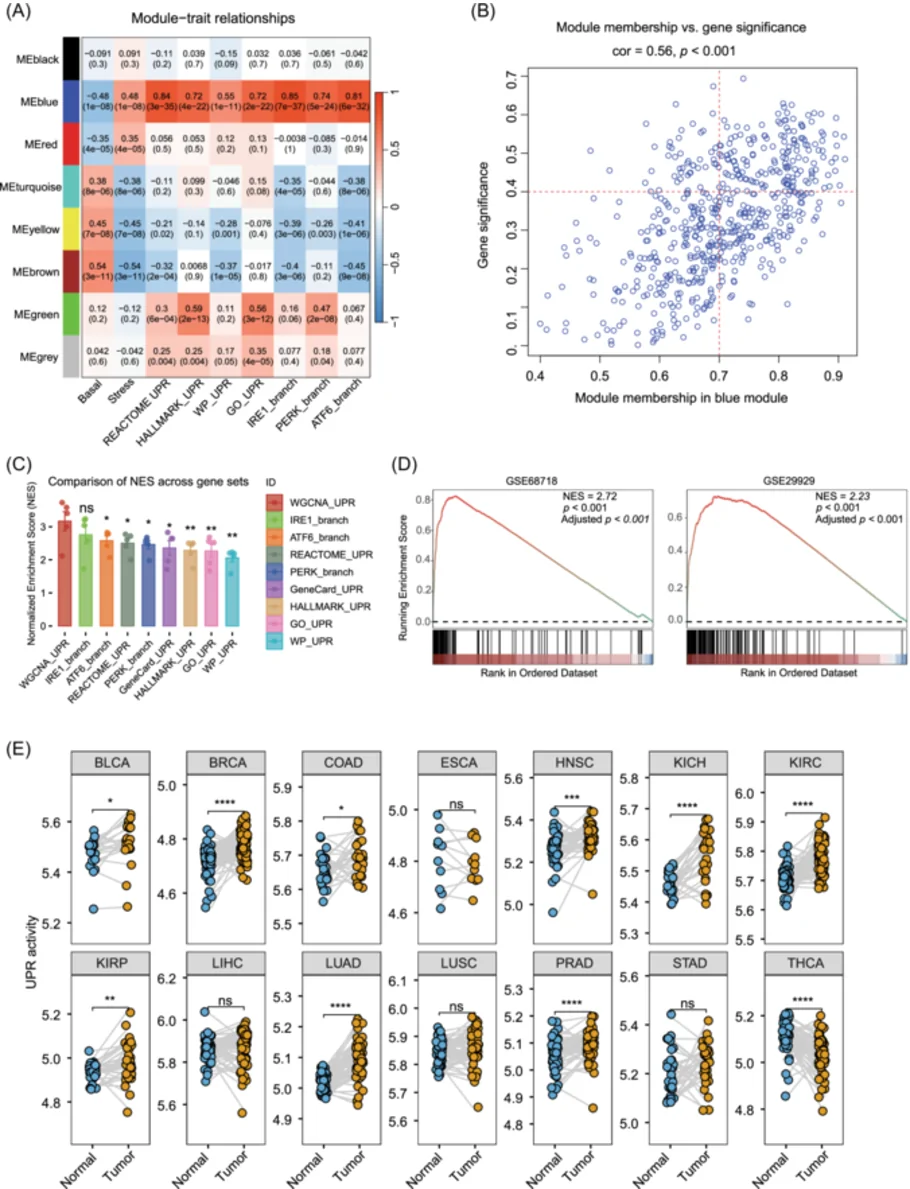
Identification and validation of the unfolded protein response (UPR) gene signature. (A) Correlation analysis between module eigengenes and phenotypes. (B) The correlation between module membership (MM) and gene significance (GS) in the blue module. (C) Mean normalized enrichment score (NES) of UPR‐related gene sets. Statistical significance was determined by a two‐tailed Student's *t*‐test. (D) Gene set enrichment analysis (GSEA) of the UPR signature in independent bulk cohorts. (E) UPR scores of 14 paired tumors and adjacent tissues in the Cancer Genome Atlas (TCGA) database. Statistical significance was determined by a two‐tailed paired Student's *t*‐test. BLCA, bladder urothelial carcinoma; BRCA, breast invasive carcinoma; COAD, colon adenocarcinoma; ESCA, esophageal carcinoma; HNSC, head and neck squamous cell carcinoma; KICH, kidney chromophobe; KIRC, kidney renal clear cell carcinoma; KIRP, kidney renal papillary cell carcinoma; LIHC, liver hepatocellular carcinoma; LUAD, lung adenocarcinoma; LUSC, lung squamous cell carcinoma; PRAD, prostate adenocarcinoma; STAD, stomach adenocarcinoma; THCA, thyroid carcinoma. * indicates *p* < 0.05, ** indicates *p* < 0.01, *** indicates *p* < 0.001, **** indicates *p* < 0.0001, and ns indicates not significant.

To evaluate the robustness of our UPR signature, we utilized seven additional datasets. These datasets included samples treated with UPR inducers TM or TG (Table S3). Next, we validated the robustness of the UPR signature in microarray datasets derived from mouse models. By analyzing 5 microarray gene expression datasets (GSE13548, GSE16157, GSE56843, GSE21979, and GSE19519) with gene set enrichment analysis (GSEA) [25], we found that the average normalized enrichment score (NES) of our UPR signature exceeded that of published UPR gene sets, indicating higher sensitivity of our UPR signature (Figure 1C). We further validated the robustness of our UPR signature across species using two mouse RNA‐seq datasets (GSE68718 and GSE29929), which yielded consistent NES results (Figure 1D). Single‐sample GSEA (ssGSEA) analysis showed that ER‐stressed samples had significantly higher scores (Figure S3A,B). The UPR score was strongly correlated with *HSPA5* (BiP/GRP78) expression (Figure S3C,D). Of note, our UPR score outperformed other publicly available gene sets in predictive AUC in the pan‐cancer dataset (Figure S4A,B).

In cancers with strong UPR activation, the NES values of paired tumor and normal tissues were generally among the highest, demonstrating that our gene set can sensitively and robustly detect UPR activation across diverse cancer types (Figure S4C). RT‐qPCR confirmed that all five rarely reported UPR‐related genes, *SUCO*, *PRRC1*, *RIOK3*, *ZFAND3*, and *IBTK*, were upregulated upon TG‐induced UPR across all three cell lines, MCF‐7, Huh7, and U2OS (Figure S4D−F). Overall, these results indicate that the UPR signature can robustly quantify UPR status across diverse cell lines, tissues, and species.

### 2.2 Delineate the UPR landscape in pan‐cancer

Using the identified UPR signature, we applied ssGSEA to over 9000 samples from 28 normal tissues based on the GTEx database, revealing tissue‐specific UPR activation patterns (Figure S5). Tissues from the reproductive system, such as the uterus, cervix, and ovaries, exhibited higher UPR scores. Conversely, tissues from the cardiovascular system, skin, and glands, including the pancreas, blood vessels, heart, and thyroid, generally displayed lower UPR scores. In parallel, we evaluated UPR status in approximately 10,000 samples of 33 tumors from the TCGA database, revealing a similar tissue preference distribution (Figure S5). Reproductive system tumors (prostate adenocarcinoma (PRAD), ovarian (OV), and uterine corpus endometrial carcinoma (UCEC)) had higher UPR scores, while tumors from the brain, skin, glands, and cardiovascular system (skin cutaneous melanoma (SKCM), acute myeloid leukemia (LAML), and thyroid carcinoma (THCA)) had relatively lower UPR scores. These results present the tissue‐specific landscape of UPR activation across normal and cancerous tissues.

We compared UPR levels between tumors and matched adjacent tissues across 14 TCGA cancer types, excluding 8 types with small sample sizes (*n* < 30). Significant differences in UPR scores were observed in 10 tumor types, notably BRCA and PRAD (Figure 1E). Among these, nine cancer types showed increased UPR activity, whereas thyroid cancer exhibited a slight decrease. Integrating TCGA and GTEx normal tissue data for a broader comparison, we found elevated UPR activity in 22 of 28 cancer types (Figure S6). These findings confirm that UPR activation is a common molecular feature in human cancers, particularly in solid tumors.

### 2.3 Cancer type‐specific association between UPR level and tumor characteristics

Although UPR activation is common in cancer, its role in tumor progression remains unclear. In 10 tumor types with distinct UPR levels, we found that UPR scores positively correlated with the MYC‐regulated network and negatively with tumor‐suppressive signatures such as ATF2‐ and PTEN‐regulated genes (Figure 2A). Furthermore, a similar correlation was observed in the remaining 23 tumor types (Figure S7). These results suggest that the induction of ER stress is often correlated with aberrant expression of oncogenes and tumor suppressor genes.

**FIGURE 2:**
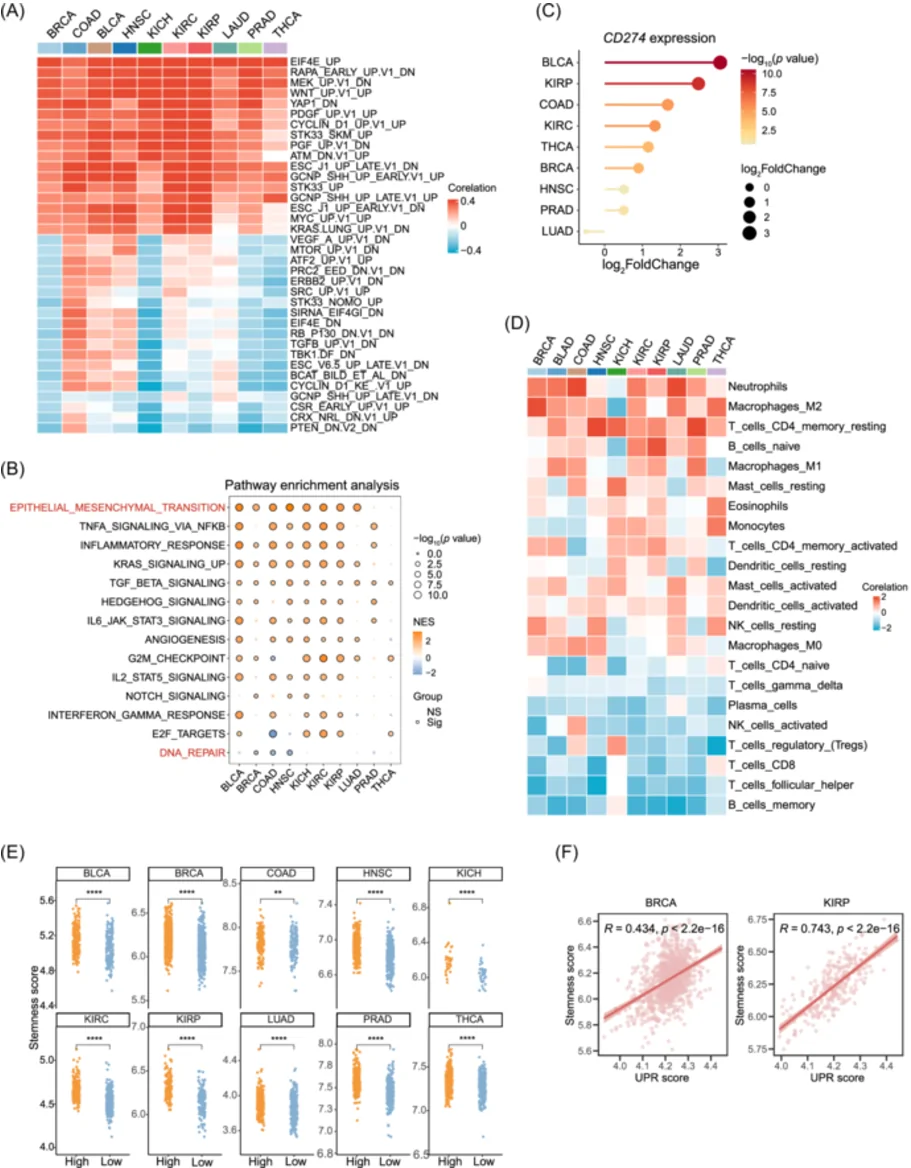
Relationship between unfolded protein response (UPR) activation and tumor progression. (A) Correlations between UPR scores and single‐sample GSEA scores of oncogenic signatures. (B) The dot plot shows the GSEA results of samples with different UPR levels. (C) The log_2_FoldChange of *CD274* expression between high‐ and low‐UPR groups. (D) Correlations between UPR scores and infiltration of distinct immune cells. (E) Differences in stemness score between the high UPR group and the low UPR group. (F) Correlations between stemness scores and UPR scores in Breast carcinoma (BRCA) and kidney renal papillary cell carcinoma (KIRP). Statistical significance was assessed using Pearson correlation. COAD, colon adenocarcinoma; BLCA, bladder urothelial carcinoma; HNSC, head and neck squamous cell carcinoma; KICH, kidney chromophobe; KIRC, kidney renal clear cell carcinoma; LUAD, lung adenocarcinoma; PRAD, prostate adenocarcinoma; THCA, thyroid carcinoma. * indicates *p* < 0.05, ** indicates *p* < 0.01, *** indicates *p* < 0.001, **** indicates *p* < 0.0001 and ns indicates not significant.

Subsequently, the samples were categorized into high‐UPR and low‐UPR groups according to the median UPR score within each cancer type (Table S4), based on which GSEA was performed. The differentially expressed genes (DEGs) in the high UPR group were also significantly enriched in the positive regulation of proinflammatory and epithelial‐mesenchymal transition (EMT) pathways in most tumors, as well as the negative regulation of the DNA repair pathway (Figure 2B). These findings link UPR activation with tumor immunity and metastasis, two critical factors affecting patient prognosis.

For tumor immunity, we set out to explore the correlation between UPR status and immune molecular characteristics. Firstly, we investigated the relationship between UPR and the expression of immune checkpoints [26]. Except for lung adenocarcinoma (LUAD), *CD274* expression was elevated in high‐UPR tumors (Figure 2C). Immune regulators such as *CD47* and *CD86* also differed between groups (Figure S8), and inhibitory molecules, including *LGALS9*, *SIRPA*, and *PDCD1*, positively correlated with UPR scores (Figure S9).

Next, using CIBERSORT, we estimated 22 immune cell types in the tumor microenvironment (TME) and found strong correlations between UPR scores and immune infiltration across tumors. UPR scores were positively associated with M2 macrophage infiltration, especially in BRCA, indicating a link between UPR activation and immune suppression (Figure 2D).

Cancer stem cells are closely associated with metastasis and drug resistance [27]. We assessed the association between UPR and tumor stemness by calculating stemness scores using a published gene set [28]. We found that high‐UPR tumors had consistently higher stemness scores than low‐UPR tumors, with strong positive correlations in multiple cancers such as BRCA and KIRC (Figure 2E,F, Figure S10), indicating a link between UPR activation and cancer stemness.

### 2.4 Single‐cell analyses reveal intra‐tumoral UPR heterogeneity

After mapping the UPR landscape across pan‐cancer, we examined UPR heterogeneity among TME cell types. Given the pronounced UPR activation in BRCA and its strong correlation with M2 macrophage infiltration and oncogenic pathway activation, we analyzed a scRNA‐seq dataset of 92,359 cells from 26 patients with BRCA [29]. Cluster analysis after the integration of all samples revealed 9 main cell lineages in BRCA (Figure S11A,B). After quantifying the UPR activity of each cell based on our UPR gene signature, we found that plasma cells and tumor cells had higher UPR scores, while T and B cells demonstrated lower UPR scores (Figure 3A,B). In the meantime, the UPR scores showed a similar distribution among BRCA patients (Figure S11C). In addition, we observed strong heterogeneity in UPR activity amongst fibroblasts. Thus, we further differentiated myofibroblasts from fibroblasts based on the established markers (Figure 3C) and found that fibroblasts exhibited higher UPR activity compared to myofibroblasts (Figure 3D).

**FIGURE 3:**
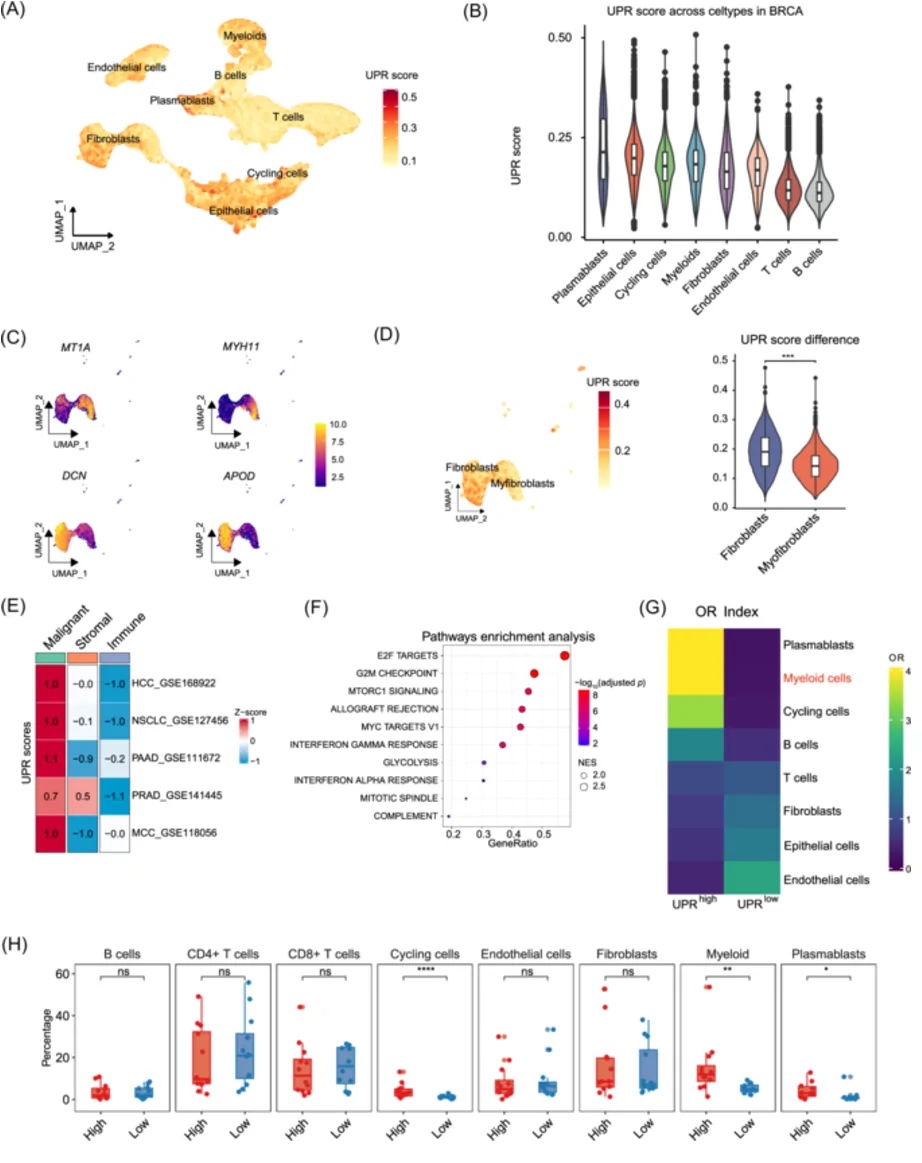
Heterogeneity of unfolded protein response (UPR) activation in the tumor microenvironment (TME). (A) The UPR scores varied across different cells in BRCA. (B) Quantification of UPR activity across different cell types. (C) Uniform Manifold Approximation and Projection (UMAP) visualization showing the expression of selected marker genes in fibroblasts. Color represents z‐score‐scaled gene expression values. (D) UPR activity difference between myofibroblast and fibroblast. (E) The average UPR scores of different cell types across tumors. (F) GSEA results of tumor cells with different UPR levels. (G) The heatmap shows the Odds Ratio (OR) of different cell types in each group. OR > 1.5 indicates that the cluster is preferred to distribute in the corresponding. (H) The distribution of distinct cell types within the TME of patients. * indicates *p* < 0.05, ** indicates *p* < 0.01, *** indicates *p* < 0.001, **** indicates *p* < 0.0001, and ns indicates not significant.

Next, we wondered whether similar intercellular heterogeneity in UPR activity existed in other tumor types. To this end, we analyzed another 5 scRNA‐seq datasets of hepatocellular carcinoma (HCC), non‐small cell lung cancer (NSCLC), pancreatic adenocarcinoma (PAAD), PRAD, and Merkel cell carcinoma (MCC). Consistently, tumor cells exhibited the highest UPR activity, while stromal cells and immune cells displayed relatively lower UPR activity (Figure 3E). Similar to BRCA, all immune cells showed the lowest UPR activity. However, plasma cells showed the highest UPR activity, exceeding that of tumor cells in HCC and NSCLC, while T and B cells had the lowest activity (Figure S12A−C). In HCC, tumor cells also exhibited significantly higher UPR activity than normal hepatocytes (Figure S12D).

### 2.5 Associations between UPR activation and transcriptomic features at the single‐cell level

Firstly, we classified the tumor cells into high and low UPR groups based on UPR activity. Differential expression analysis of tumor cells revealed significant enrichment of pathways related to cell cycle and proliferation, metabolic reprogramming, and inflammatory responses (Figure 3F), broadly consistent with the bulk transcriptomic results. To further explore the impact of UPR activation on the TME, we classified the 24 BRCA samples into high UPR and low UPR groups based on their UPR scores using the matched bulk RNA‐seq data. According to the odds ratio (OR) analysis, the plasmablasts and myeloid cells showed a strong distribution preference in the high UPR group (Figure 3G). Meanwhile, we observed increased infiltration of myeloid cells, plasma cells, and cycling cells in patients of the high UPR group (Figure 3H). These findings suggest that the UPR activity of malignant cells may impact the infiltration of immune cells, in particular myeloid cells, in the TME of BRCA.

### 2.6 Characteristics of myeloid cell subpopulation in the TME

Myeloid cells are one of the most abundant immune cells within the TME, closely associated with prognosis and therapy resistance [30]. Given the significant increment in myeloid cell infiltration in the high UPR group, we re‐clustered myeloid cells into 10 subpopulations, including 6 macrophage clusters (Macro 1−6), 1 monocyte cluster, 2 dendritic cells (DC1 and DC2), and cycling cells (Figures 4A, S13A). Our results showed that Macro3 and Macro4 demonstrated the highest level of UPR activity compared to other subpopulations (Figure 4B). Then, we compared the proportions of these cell clusters between the high and low UPR groups and revealed an increased abundance of Macro3 and Macro4 cells in the high UPR group, as well as a decreased abundance of DC1 (Figure 4C).

**FIGURE 4:**
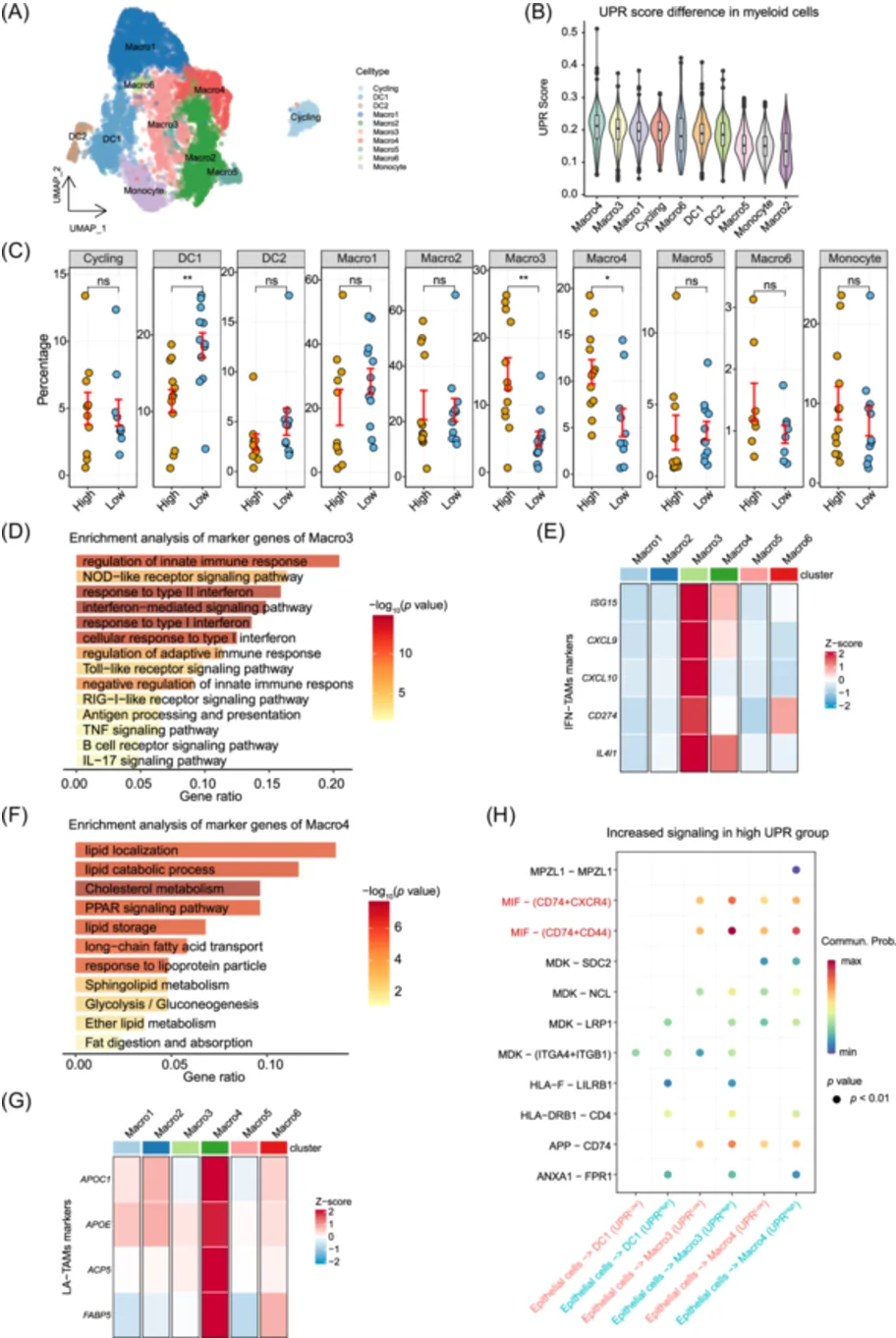
Tumor cells may facilitate Macro3 and Macro4 infiltration via the MIF signaling pathway. (A) UMAP shows 10 myeloid clusters. (B) The feature plot shows unfolded protein response (UPR) activity varied across different cells. (C) The proportions of myeloid clusters in the TME of patients with different UPR levels. (D) Bar plot showing the enrichment analysis of marker genes for Macro3. (E) Z‐score‐normalized expression levels of interferon‐associated tumor‐associated macrophage (IFN‐TAM) markers in myeloid cells. (F) Bar plot showing the enrichment analysis of marker genes for Macro4. (G) Z‐score‐normalized expression levels of lipid‐associated tumor‐associated macrophage (LA‐TAM) markers in myeloid cells. (H) Tumor cells may frequently communicate with Macro3 and Macro4 through MIF signals in the high‐UPR group. * indicates *p* < 0.05, ** indicates *p* < 0.01, and ns indicates not significant.

To gain insight into the biological features of Macro3 and Macro4, we performed enrichment analysis of different clusters (Figure S13B−E). Upregulated DEGs in Macro3 were predominantly associated with response to interferon (Figure 4D). In comparison to other myeloid cells, Macro3 demonstrated heightened levels of interferon‐regulated genes, such as *ISG15*, *CXCL9*, *CXCL10*, and *IL4I1*, as well as immune checkpoint molecule *CD274* (Figure 4E). Meanwhile, upregulated DEGs in Macro4 were predominantly associated with lipid metabolism (Figure 4F). Macro4 highly expressed lipid‐related genes, including *APOC1*, *APOE*, *ACP5*, and *FABP5*, compared to other cells (Figure 4G), and exhibited the highest lipid metabolism activity among all macrophages (Figure S13F). Lastly, the high UPR group exhibited higher communication frequency and intensity between tumor cells and DC1, Macro3, and Macro4 cells (Figure S14). According to the intercellular communication analyses, we discovered that in tumors with high UPR activity, tumor cells frequently engaged in communication with Macro3 and Macro4 through the MIF signaling pathway (Figure 4H), indicating that tumor cells with activated UPR may enhance the infiltration of Macro3 and Macro4 cells through MIF signaling.

### 2.7 Predicting immunotherapy response according to UPR score

Motivated by the link between TME remodeling and UPR activation, we assessed whether UPR scores could predict immunotherapy response by comparing tumor cell UPR scores in paired samples before and after treatment. Firstly, we analyzed a single‐cell dataset of an immunotherapy‐sensitive BRCA mouse model. As expected, the UPR scores of tumor cells decreased after anti‐PD1/anti‐CTLA4 treatment in tumor cells (Figure 5A,B), whereas certain immune cell populations, such as B cells and NK cells, exhibited an increasing trend (Figure S15A). On the other hand, in the case of immunotherapy‐resistant MCC patients, the UPR score significantly increased after treatment (Figure 5C,D), whereas no significant change was observed in the UPR scores within the TME (Figure S15B). Subsequently, in five BRCA cohorts, UPR scores positively correlated with immunosuppressive molecules (*PDCD1*, *BATF*, *ENTPD1*) and cytokines (*IL4*, *IL13*, *IL1A*, *IFNA2*), but negatively with immunostimulatory molecules such as *GZMK*, *CST7*, and *GZMA* (Figure 5E). These findings suggest that higher UPR activation is associated with impaired antitumor immunity and that tumors with elevated UPR scores tend to be resistant to immunotherapy.

**FIGURE 5:**
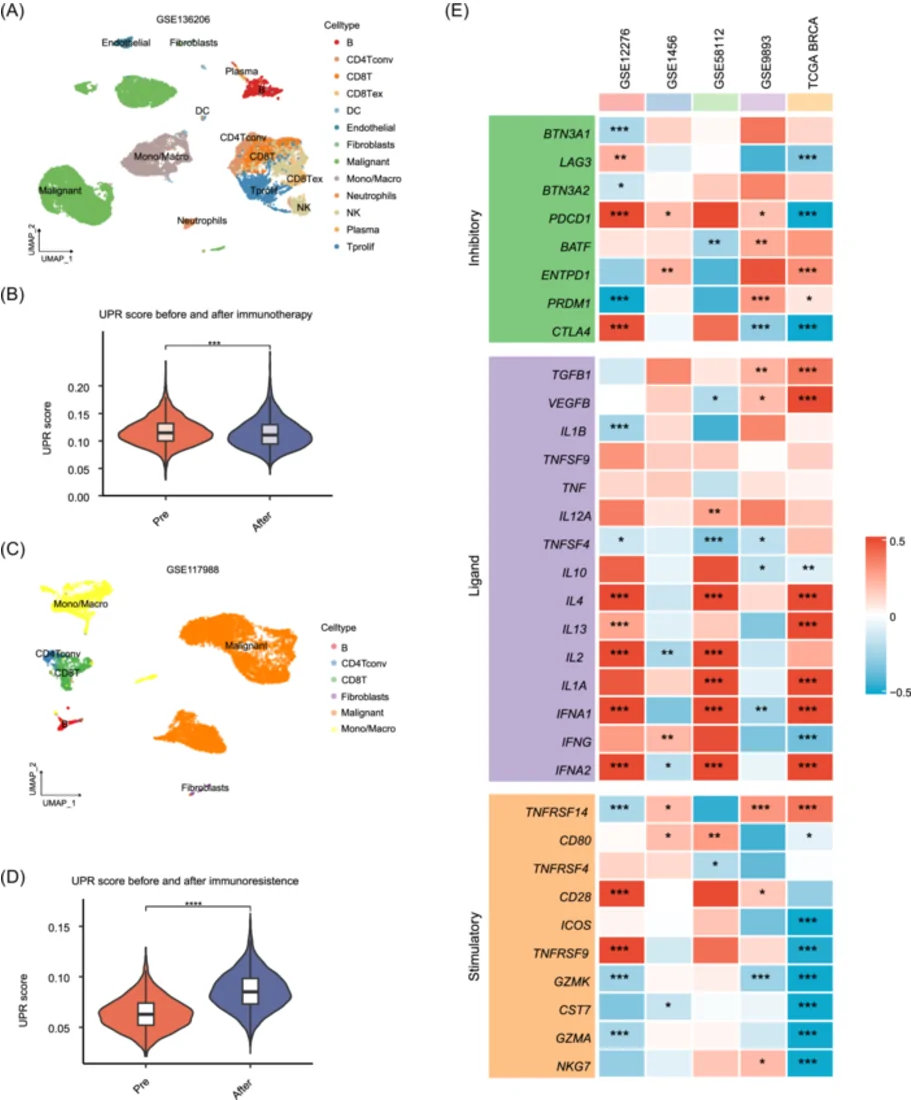
Correlations between unfolded protein response (UPR) activation and immunotherapy response. (A) UMAP plot showing the main cell types in single‐cell datasets of BRCA mouse models receiving immunotherapy. (B) Post‐treatment changes in UPR levels of tumor cells from responders and non‐responders in BRCA mouse models. (C) UMAP plot showing the main cell types in single‐cell datasets of Merkel cell carcinoma (MCC) patients receiving immunotherapy. (D) Post‐treatment changes in UPR levels of tumor cells from responders and non‐responders in MCC patients. Statistical significance was assessed by the two‐tailed Wilcoxon rank‐sum test. (E) Heatmap shows the associations between UPR scores and expression of immunostimulatory, inflammation‐related, and immunosuppression‐related molecules in BRCA cohorts. Correlations were calculated using Spearman's rank correlation coefficient. *** indicates *p* < 0.001, **** indicates *p* < 0.0001.

### 2.8 UPR status to predict BRCA prognosis

Lastly, to explore the translational value of our UPR signature, we employed three machine‐learning algorithms to identify prognostic hub genes (Figure 6A). We collected bulk transcriptomic data from 5 BRCA cohorts with 1722 patients, where the TCGA‐BRCA cohort was used as the training dataset, and the remaining cohorts served as independent validation datasets. Through implementing the Least Absolute Shrinkage and Selection Operator (LASSO), eXtreme Gradient Boosting (XGBoost), and random forest with Boruta (RFB), we identified 24, 37, and 10 hub genes associated with survival, respectively (Table S5). Subsequently, we constructed a multiple Cox regression model using three commonly observed genes, *SHMT2*, *KDELR2*, and *SLC1A4*, and confirmed by RT‐qPCR that their expression was significantly upregulated upon TG treatment in MCF7 cells (Figure S16A). Finally, we defined the UPR predictor as follows: UPR predictor = 0.37306 × (expression of *SHMT2*) + 0.38118 × (expression of *KDELR2*) − 0.14916 × (expression of *SLC1A4*).

**FIGURE 6:**
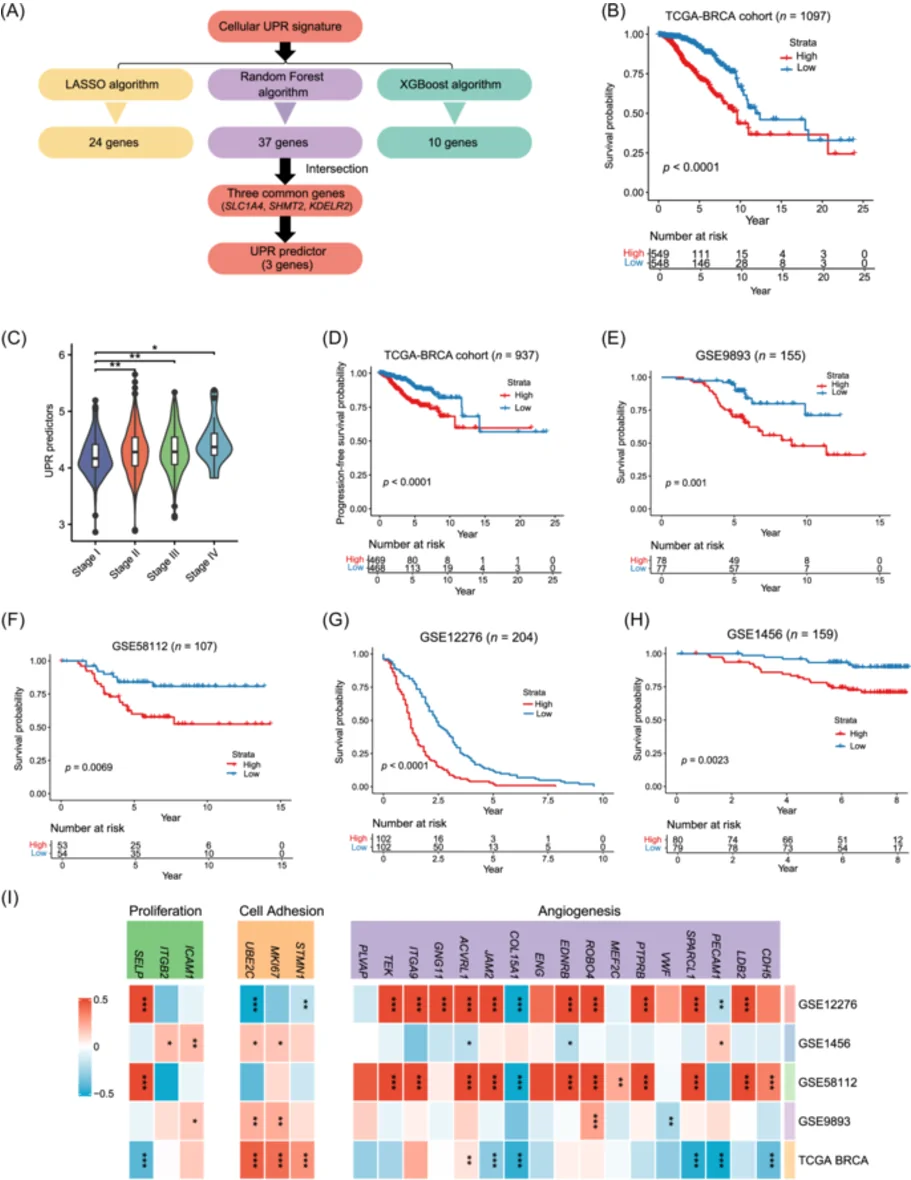
Unfolded protein response (UPR)‐related genes are potential prognostic predictors of breast cancer patients. (A) The overall strategy for selecting three hub genes in the UPR signature to predict the survival of BRCA patients. (B) Kaplan–Meier curves showing overall survival of patients with high and low UPR predictors in the TCGA‐BRCA cohort. (C) Violin plot showing the distribution of UPR predictors among patients with different clinical stages in the TCGA‐BRCA cohort. (D) Kaplan–Meier curve of progression‐free survival (PFS) for patients with high and low UPR predictor scores in the TCGA‐BRCA cohort. (E–H) Kaplan–Meier survival analysis of patients stratified by high and low UPR predictor scores in four independent BRCA cohorts, including GSE9893 (E), GSE58112 (F), GSE12276 (G), and GSE1456 (H). Differences in survival were assessed by the log‐rank test. (I) Heatmap shows the associations between UPR scores and expression of genes associated with proliferation, angiogenesis, and cell adhesion in five BRCA cohorts. Correlations were calculated using Spearman's rank correlation coefficient. * indicates *p* < 0.05, ** indicates *p* < 0.01, *** indicates *p* < 0.001.

To validate the prognostic value of the UPR predictor, patients were divided into high‐ and low‐risk groups based on the median of the UPR predictors. As expected, individuals with elevated UPR predictors showed worse prognosis and more advanced pathological stages (Figure 6B−D). After adjusting for covariates such as age, sex, and race, higher UPR scores remained associated with poorer prognosis (Figure S16B). Likewise, higher UPR scores were generally associated with worse survival outcomes in the 4 validation cohorts (GSE9893, GSE58112, GSE12276, and GSE1456) (Figure 6E−H). The UPR predictor demonstrated promising prognostic performance with AUCs of 0.62–0.76 in the validation sets and outperformed representative previously published models (Figure S16C−F). Besides BRCA, the UPR predictor also showed prognostic potential in 10 other tumor types, including adrenocortical carcinoma (ACC) and bladder urothelial carcinoma (Figure S17). In addition, the UPR scores were positively correlated with the expression of genes involved in tumor proliferation, angiogenesis, and extracellular matrix remodeling, such as *TEK*, *ITGA9*, *EDNRB*, *SELP*, and *ICAM1* (Figure 6I). Together, these data demonstrate the power of our UPR signature in predicting patient prognoses.

## 3 DISCUSSION

UPR activation may represent one of the common molecular characteristics in human cancers, particularly in solid tumors [31]. Accumulating evidence has implicated critical roles of the UPR pathways in human cancer [32], underscoring the need for a comprehensive pan‐cancer evaluation of the UPR landscape and its relationships with key tumor characteristics [33]. To this end, we developed a UPR signature and assessed UPR activity utilizing UPR scores derived from the ssGSEA algorithm. Of note, our UPR score serves as a quantitative indicator of UPR activation levels, rather than directly reflecting stress or baseline level per se. Therefore, comparing the score between cancerous and the corresponding normal tissues would help to define UPR activation status specific to a particular cancer or tissue type. This enabled us to map the intra‐ and inter‐tumoral UPR landscape and to reveal the heterogeneity of UPR activity in different tissues and cell types. Furthermore, we discovered a strong correlation between UPR activation and crucial genomic and immune features in cancer. In addition, with the use of three machine learning algorithms, we proposed a promising prognostic biomarker for BRCA from the perspective of UPR. These results presented an efficient method for assessing UPR activity in cancer and highlighted the translational significance of UPR in cancer.

To accurately evaluate the UPR status, we developed a specific UPR signature across the range of pan‐cancer. When developing the UPR signature, common features of different tissue types, cell lines, and inducers were taken into consideration. It provided the same criteria for comparing UPR activity across various tumor types. Based on the signature, we assessed UPR activation in pan‐cancer and revealed the intra‐ and inter‐tumoral heterogeneity of UPR. We noticed that tissue and cell origin were vital factors influencing UPR activity. For example, relatively higher basal UPR activity is prevalent in epithelial cells of vigorous secretory demand, such as the breast and prostate tissues, and UPR activity remained high in their respective malignancies. Although this study delineated a pan‐cancer landscape of UPR activation based on UPR scores, systematically assessing features such as tumor aggressiveness and therapeutic sensitivity, which are influenced by multiple factors, remains challenging. Future studies should integrate multi‐omics data and experimental validation to further investigate the differences between tumors with high and low UPR activation in terms of aggressiveness and therapeutic sensitivity. Regarding the oncogenic network, we found that UPR activity was significantly correlated with EMT and tumor stemness score. A couple of recent studies have functionally linked UPR activation with EMT [34] and cancer cell stemness [35]. Mechanistically, activation of UPR pathways such as PERK‐eIF2α‐ATF4 and IRE1α‐XBP1 promotes cancer cell plasticity and stemness by upregulating transcription factors including HIF1α, MYC, SOX2, and NANOG, thereby coupling stress adaptation with transcriptional reprogramming to sustain tumor‐initiating capacity [13, 36, 37, 38]. However, the detailed underlying mechanisms as to how UPR activation leads to enhanced metastasis remain largely unclear.

Furthermore, we investigated the association between UPR activation and tumor immunity and unveiled a critical link between UPR and tumor immunosuppression. For instance, UPR scores demonstrated a positive correlation with M2‐macrophage infiltration and immune checkpoint expression. Further analysis showed that UPR activation may promote the infiltration of macrophages, particularly those of Macro3 and Macro4. These two subtypes of macrophages exhibited characteristics of interferon‐primed TAMs and lipid‐associated TAMs, respectively, which were known to exert suppressive functions on anti‐tumor immunity [30, 39, 40]. These findings are in line with the established important roles of macrophages in tumors [41, 42].

Importantly, we observed that UPR‐related genes showed significant clinical implications for immunotherapy response and patient prognosis. The distinct variations in UPR scores between responders and non‐responders of immunotherapy underscore the potential of UPR as a predictive marker for immunotherapy efficacy. Notably, recent studies also show that stress‐response‐related programs are closely associated with tumor treatment outcomes, further supporting their value in predicting immunotherapy benefit [43]. Yet, this observation should be validated in additional immunotherapy cohorts of larger sample sizes. Moreover, it would be important to evaluate how UPR scores complement or provide alternatives to currently approved biomarkers such as PD‐L1 expression, tumor mutational burden, and microsatellite instability. Future studies may further establish assessment frameworks to determine the added predictive value of integrating UPR with these established biomarkers in guiding immunotherapy. Taking BRCA as an example, we identified three hub genes by adopting different machine‐learning algorithms that predicted patient survival. Compared to the representative previous prognostic models [44, 45, 46], our prognosis model derived from the perspective of UPR demonstrated robust power in predicting outcomes for BRCA patients across multiple datasets. The expression of these genes is abundant and easily detectable, endorsing their great potential for clinical translation. Specifically, these genes could be measured using accessible platforms such as immunohistochemistry, multiplexed protein assays, or targeted transcriptomic panels. Cohort‐specific thresholds could be established to stratify patients by risk, enabling potential application in prognosis assessment and therapeutic response monitoring.

Despite these advancements, the present study still has several limitations. Firstly, the UPR signature we developed only enrolled the UPR activation‐related genes, while the inhibition‐related genes were neglected. Future studies need to combine UPR inhibition‐related genes to assess UPR activity more rigorously and accurately. Secondly, our UPR signature assesses overall UPR activity instead of a detailed evaluation of each branch; while some of the genes in our UPR signature are not exclusive to ER stress. Thirdly, although scRNA‐seq technology can resolve the heterogeneity of the TME at the level of individual cells, its sequencing depth is relatively low, resulting in drop‐out phenomena, which further impact the accuracy of UPR score evaluation. Therefore, when applying our UPR signature to interpret scRNA‐seq data with relatively poor sequencing quality, the accuracy of the UPR score should be double‐checked. Lastly, our bioinformatics analyses only provided correlational, rather than causal, relationships between UPR activation and cancer characteristics. Experimental investigations are warranted to determine the causal effect as well as the intrinsic molecular mechanisms. Currently, a variety of small molecules targeting key nodes of the UPR are under preclinical and clinical development [16]. One of the future directions ahead lies in the rapid and accurate evaluation of branch‐specific activation of UPR in different cancers, which would facilitate the precise identification of patient subtypes that may benefit from specific therapeutic strategies.

In summary, the present study provides a viable method for assessing UPR activity across human cancers and underscores the clinical relevance of UPR activation in the advancement of cancer.

## 4 CONCLUSION

In this study, we systematically evaluated UPR activity across human cancers and revealed its substantial heterogeneity across tumor types and cellular contexts, along with strong associations with key genomic and immune features. We also identified a UPR‐related prognostic biomarker with potential clinical utility in breast cancer. Collectively, these findings deepen our understanding of the role of UPR in cancer and provide a valuable reference for future investigations into its mechanistic and therapeutic relevance.

## 5 METHODS

### 5.1 Data collection

Gene expression matrix and related information in the GTEx and TCGA databases were collected from UCSC Xena (http://xena.ucsc.edu/public/). scRNA‐seq and RNA‐seq data were collected from the Gene Expression Omnibus (GEO) database (https://www.ncbi.nlm.nih.gov/geo/). To build an integrated resource of UPR transcriptomic features, we conducted a comprehensive search of literature and databases to collect published UPR transcriptomic profiles. Subsequently, we curated a collection comprising 130 UPR‐related samples from 16 bulk RNA‐seq datasets (GSE84636, GSE66556, GSE196634, GSE169584, GSE153557, GSE148802, GSE146840, GSE145108, GSE139391, GSE131048, GSE129757, GSE108212, GSE200205, GSE217305, GSE226549, and GSE227811), and downloaded FASTQ files from the official websites. Seven UPR‐related datasets (GSE68718, GSE29929, GSE13548, GSE16157, GSE56843, GSE21979, and GSE19519) served as validation datasets to estimate the robustness of the UPR signature. Four cohorts containing survival information (GSE12276, GSE1456, GSE58112, GSE9893) were used to verify the robustness of the survival prediction model. Five scRNA‐seq datasets (GSE168922, GSE111672, GSE127456, GSE141445, GSE118056) were used to explore cell type heterogeneity at the UPR level and its crosstalk in the TME. Two scRNA‐seq datasets (GSE136206, GSE117988) were used to predict the immunotherapy response.

### 5.2 Analysis of bulk RNA‐seq data

For the collected UPR‐related FASTQ files, we employed a standardized pipeline for further processing: (1) Evaluation of the overall quality of sequencing reads using FastQC (v0.11.9). (2) Alignment to the reference human genome (GRCh38) and generation of count matrices using Salmon (v1.4.0). A summary of sample and dataset characteristics is provided in Table S1. (3) The raw gene expression count matrix was normalized to counts per million (TPM) and then log_2_‐transformed as log_2_(TPM + 0.1). The “ComBat” function in the R package “sva” (v3.48.0) was employed to remove batch effects of data from different sources [47].

### 5.3 Weighted correlation network analysis (WGCNA)

We utilized the RNA‐seq expression matrix to generate a co‐expressed RNA network using the R package “WGCNA” (v1.72.1). WGCNA was performed on all samples, including both basal and stress conditions, to ensure sufficient sample size and expression variability for robust and stable network construction, rather than restricting the analysis to stress samples only. We retained all mRNAs for further analysis. Instead of using all expressed genes, we focused on the top 5000 genes ranked by median absolute deviation. To determine the appropriate soft‐thresholding power (*β*) in WGCNA, we evaluated a series of candidate β values using the “pickSoftThreshold” function. For each candidate β, the scale‐free topology fit index (signed *R* ^2^) and mean connectivity were calculated to assess whether the resulting network satisfied the scale‐free topology criterion. The smallest *β* value at which the signed *R* ^2^ reached 0.85 while maintaining relatively high mean connectivity was selected as the optimal soft‐thresholding power. Based on these criteria, *β* = 12 was chosen for subsequent network construction. The weighted adjacency matrix was converted into a topological overlap matrix (TOM) to generate the corresponding dissimilarity (1‐TOM). We applied the dynamic tree‐cutting method to identify modules and selected the modules with the highest correlation to UPR for further analysis. We constructed the UPR gene signature based on the following criteria: (1) inclusion of genes within the module most closely associated with the ER stress state, and (2) genes with a gene significance (GS) > 0.4 and module membership (MM) > 0.7. A total of 579 genes were highly associated with the ER stress state, and 159 genes were ultimately included in the UPR signature.

### 5.4 UPR score calculation

UPR activation levels were quantified using the ssGSEA method implemented within the R package “GSVA” (v1.48.3) [48]. Subsequently, tumor samples were categorized into two groups, namely high‐ and low‐UPR groups, based on the median UPR scores. We employed the R package “AUCell” (v1.22.0) for the scRNA‐seq dataset to calculate each cell's UPR activity. Within the same cell type, the top 5% of cells with the highest UPR scores were designated as the high‐UPR group, while the bottom 5% of cells with the lowest UPR scores were classified as the low‐UPR group. This intra‐class threshold was chosen to capture the extreme ends of UPR activation while ensuring adequate cell numbers for comparison.

### 5.5 Immune cell infiltration estimation

The relative abundance of 28 immune cell types in TCGA cohorts was quantified using the CIBERSORT algorithm [49] implemented via the R package “IOBR” (v0.99.9). To assess the potential relationship between UPR activation and immune cell infiltration, Spearman's rank correlation analysis was performed between the UPR scores of each sample and the estimated immune cell fractions obtained from CIBERSORT. The resulting correlation matrix was subsequently visualized as a heatmap using the R package “ComplexHeatmap” (v2.16.0), allowing intuitive inspection of positive and negative associations between UPR activity and specific immune cell types.

### 5.6 Analysis of scRNA‐seq data

The count matrix of BRCA scRNA‐seq data (GSE176078) was imported into the R package “Seurat” (v4.0.4). To ensure quality, low‐quality cells were identified on the following criteria: (1) nFeatures < 200 or > 5000 and (2) mitochondrial gene ratio > 15%. After filtering, a total of 92,359 high‐quality cells remained for subsequent analyses. The R package “harmony” (v1.2.0) was used for data normalization, correction of batch effects arising from patient origin, cluster identification, and visualization [50]. Differential expression analysis was performed by first aggregating highly similar single cells into Metacell‐like SuperCells using the “SuperCell” (v1.1) R package with gamma = 20, k.knn = 5, the top n.pc = 10, and n.var.genes = 1000. DEGs between high‐ and low‐UPR groups were then identified at the SuperCell level using the FindMarkers function from the Seurat R package, with adjusted *p* < 0.05 and |log2FC| > 1 considered significant, followed by pathway enrichment analysis using the “clusterProfiler” (v4.8.3) R package. This approach reduces single‐cell noise, minimizes false positives, and mitigates false negatives arising from pseudobulk aggregation. This approach reduces single‐cell noise, minimizes false positives in FindMarkers results, and mitigates false negatives that can arise from pseudobulk aggregation [51]. Using the R package “Cellchat” (v1.6.1) [52] for cell and cell communication analysis, utilizing the count matrix as input. Significant interaction pairs were determined based on *p* < 0.05.

### 5.7 Construction of the UPR predictor for BRCA

The essential prognosis‐related genes in the UPR signature were identified using three machine‐learning algorithms: LASSO, RFB, and XGBoost. This process is completed using the R packages “glmnet” (v4.1.8), “randomForestSRC” (v3.3.0), and “xgboost” (v1.7.7.1). Finally, the three common genes (*SHMT2*, *KDELR2*, and *SLCA4*) were used to construct a multiple Cox regression model. The UPR predictor was developed using 5 BRCA cohorts consisting of 1562 patients. The training dataset utilized TCGA BRCA, while the validation datasets included GSE9893, GSE58112, GSE12276, and GSE1456. Samples were classified into high and low groups based on the median values of the UPR predictors.

### 5.8 Survival analysis

Patients were categorized into high‐UPR and low‐UPR groups based on the median UPR predictors. The R package “survival” (v3.5.5) was utilized to conduct the survival analyses. Kaplan‐Meier survival curves were used to estimate the survival differences between the two groups. We employed the log‐rank test to determine significant differences, and *p* < 0.05 was regarded as statistically significant. ROC analysis was performed using the R package “survivalROC” (v1.0.3.1).

### 5.9 Cell culture

The human hepatocellular carcinoma cell line Huh7, breast adenocarcinoma cell line MCF‐7, and osteosarcoma cell line U2OS were cultured in medium supplemented with 10% fetal bovine serum (FBS) and a dual antibiotic solution (penicillin at 100 U/mL and streptomycin at 100 U/mL), and maintained in a humidified incubator at 37°C. Upon reaching 90% confluence, the cells were removed from the incubator. For the experimental group, thapsigargin (TG) was added at a final concentration of 30 nM, while the control group was treated with DMSO alone. After 3 h of treatment, the cells were harvested for subsequent experiments, including RNA extraction.

### 5.10 RNA extraction and RT‐qPCR

Total RNA was extracted from MCF‐7, Huh7, and U2OS cells using the Trizol method, and reverse‐transcribed into cDNA using the Vazyme reverse transcription kit (Cat# R323‐01, Vazyme Biotech Co., Ltd). The resulting cDNA was stored at −20°C for subsequent experiments.

Primers were designed using the NCBI platform, with GAPDH as the internal reference gene and the cDNA from the three cancer cell lines as the template. Quantitative PCR was performed on an Applied Biosystems 7500 system using ChamQ Universal SYBR qPCR Master Mix (Cat# Q711‐02, Vazyme Biotech Co., Ltd.) under the following cycling conditions: initial denaturation at 95°C for 10 min; 45 cycles of 95°C for 30 s, 60°C for 30 s, and 72°C for 30 s. Cycle threshold (Ct) values were exported, and relative gene expression levels were calculated using the 2^−ΔΔCt^ method. Primer sequences are listed in Table S6.

### 5.11 Statistical analysis

All analyses were completed in R (v4.1.1) (https://www.r-project.org). Statistical analyses were conducted to compare continuous variables between the two groups using either the Student's *t*‐test or the Wilcoxon test. Differences between the three groups were compared using univariate analysis of variance or the Kruskal–Wallis test. Correlations among normally distributed variables were evaluated using Pearson correlation analysis, while correlations among non‐normally distributed variables were assessed using Spearman correlation analysis. All statistical tests were two‐sided, and *p* < 0.05 was regarded as statistically significant.

## AUTHOR CONTRIBUTIONS

**Xinyu Yang and Faming Zhao**: Data curation; investigation; formal analysis. **Jing Yang**: Software; data curation; visualization. **Xinran Xia**: Investigation; validation. **Liwei Chen**: Methodology; software. **Peng Zeng**: Writing — original draft. **Liang Chen**: Project administration. **Yanxia Hu**: Conceptualization; writing — review and editing. **Xia Sheng**: Supervision; funding acquisition; writing — review and editing. All authors have read the final manuscript and approved it for publication.

## CONFLICT OF INTEREST STATEMENT

The authors declare no conflicts of interest.

## ETHICS STATEMENT

No animals or humans were involved in this study.

## Supporting information


**Figure S1.** Evaluation of batch effects across datasets using PCA.
**Figure S2.** WGCNA analysis.
**Figure S3.** Validation of the UPR signature.
**Figure S4.** Performance and validation of UPR gene sets.
**Figure S5.** UPR landscape across normal (GTEx) and tumor (TCGA) tissues.
**Figure S6.** Comparison of UPR scores between tumors and normal tissues.
**Figure S7.** Correlation between UPR scores and oncogenic signatures.
**Figure S8.** Expression differences of immunosuppressive molecules between the high‐ and low‐UPR groups.
**Figure S9.** Correlation between UPR scores and immune checkpoint expression.
**Figure S10.** Correlation between stemness scores and UPR scores in pan‐cancer.
**Figure S11.** Single‐cell transcriptomics reveals heterogeneity in UPR activity within the TME.
**Figure S12.** UPR activity difference in TME across different tumors.
**Figure S13.** Characteristics of different subpopulations of macrophages.
**Figure S14.** Analysis of intercellular communication.
**Figure S15.** UPR activity of immune cells in immunotherapy‐treated patients.
**Figure S16.** Evaluation of the prognostic models.
**Figure S17.** Kaplan‐Meier curves of overall survival stratified by UPR predictors.

**Table S1.** Summary of datasets used to develop the pan‐cancer unfolded UPR signature.
**Table S2.** List of genes comprising the UPR gene signature.
**Table S3.** Sample information of the validation datasets.
**Table S4.** Median UPR scores across different cancer types.
**Table S5.** Key UPR‐related features identified by different machine learning algorithms.
**Table S6.** List of primers used for quantitative real‐time PCR (RT‐qPCR).

## Data Availability

Public data used in this work are available from the TCGA (https://portal.gdc.cancer.gov/), GTEx (https://gtexportal.org/), and GEO (https://www.ncbi.nlm.nih.gov/geo/) databases. All data generated throughout this study are included within the manuscript and the supplementary materials. All analysis scripts are publicly available on the GitHub website (https://github.com/Yyyxya/UPR). Supplementary materials (tables, figures, graphical abstract, slides, videos, Chinese translated version, and updated materials) may be found in the online DOI or iMeta Science http://www.imeta.science/imetaomics/.
