# Supplementary material for: Comprehensive assessment of unfolded protein response and its association with tumor progression in pan‐cancer
Source: IMetaOmics. 2026 Feb 20;3(1):e70084. doi: 10.1002/imo2.70084 (PMC13674402; doi:10.1002/imo2.70084)
Supplement: Supplementary file 1 [file IMO2-3-e70084-s001.docx]

**Supplementary information to**

**Comprehensive assessment of unfolded protein response and its association with tumor progression in pan-cancer**

**Running title:** Unfolded protein response and tumor progression in pan-cancer.

Xinyu Yang^1,2,3#^, Faming Zhao^1,2,3#^, Jing Yang^3#^, Xinran Xia^3^, Liwei Chen^3^, Peng Zeng^3^, Liang Chen^1^*, Yanxia Hu^3^*, Xia Sheng^3^*

^1^Department of Urology, Tongji Hospital, Tongji Medical College, Huazhong University of Science and Technology, Wuhan 430030, China

^2^School of Public Health, Tongji Medical College, Huazhong University of Science and Technology, Wuhan 430030, China

^3^Institute of Biomedical Research, School of Life and Health Sciences, Hainan University, Haikou 570228, China

#These authors contributed equally: Xinyu Yang, Faming Zhao, Yang Jing.

Correspondence: chenliang@tjh.tjmu.edu.cn (Liang Chen); huyanxia2024@hainanu.edu.cn (Yanxia Hu); xsheng@hainanu.edu.cn (Xia Sheng).

**Figure S1 Evaluation of batch effects across datasets using PCA.** (A) PCA plots of samples from different sources and biological states before removing batch effects. (B) After batch effect removal, the PCA plots of samples from various sources and biological conditions.

**Figure S2 WGCNA analysis.** (A) The network analysis involves studying the impact of different soft-threshold powers on the topology of the network. The left panel displays the scale-free topology fit index for each power, while the right panel shows the mean connectivity for each power. (B) The heatmap presents the adjacency of eigengenes within modules. (C) Pathway enrichment analysis of genes within the blue module. (D) Correlation analysis between module eigengenes and phenotypes after excluding central nervous system tumor cell lines. (E) Overlap between the original module (Mblue1) and the module identified after excluding central nervous system tumor cell lines (Mblue2).

**Figure S3 Validation of the UPR signature.** (A and B) ssGSEA of samples in different biological states. (C and D) Correlations between *HSPA5* expression and UPR scores. ** indicates *p* < 0.01, *** indicates *p* < 0.001, **** indicates *p* < 0.0001, and ns indicates not significant.

**Figure S4 Performance and validation of UPR gene sets.** (A) Receiver operating characteristic (ROC) curves showing the performance of gene sets in predicting UPR states across multiple cancer types. (B) Area under the ROC curve (AUC) values for each gene set in predicting UPR states. (C)Heatmap depicting the ranking of NES scores for various gene sets in five cancers exhibiting strong UPR activation. (D-F) RT-qPCR analysis showed that TG-induced UPR significantly upregulates the expression of the rarely reported genes *SUCO, PRRC1, RIOK3, ZFAND3,* and *IBTK* in MCF-7 (D), U2OS (E), and Huh7 (F) cells. Statistical significance was determined by two-tailed *t*-test:* indicates *p* < 0.05, ** indicates *p* < 0.01, *** indicates *p* < 0.001, **** indicates *p* < 0.0001.

**Figure S5 UPR landscape across normal (GTEx) and tumor (TCGA) tissues.** UPR landscape across 28 normal tissue types from GTEx (left) and 33 tumor types from TCGA (right). The UPR activity was independently assessed in non-cancerous and malignant samples to capture tissue-specific patterns of UPR activation. The percentages represent the relative UPR activity of each tissue, normalized to the highest UPR score (set as 100%). AG, Adrenal gland; AT, Adipose tissue; BM, Bone marrow; BV, Blood vessel; SG, Salivary gland; SI, Small intestine.

**Figure S6 Comparison of UPR scores between tumors and normal tissues.** UPR scores of tumors and normal tissues in the TCGA and GTEx database. Statistical significance was determined by the two-tailed Wilcoxon rank-sum test. * indicates *p* < 0.05, ** indicates *p* < 0.01, *** indicates *p* < 0.001, **** indicates *p* < 0.0001, and ns indicates not significant.

**Figure S7 Correlation between UPR scores and oncogenic signatures.** Correlations between UPR scores and ssGSEA scores of oncogenic signatures in cancers. Associations were assessed using Spearman’s rank correlation coefficient.

**Figure S8 Expression differences of immunosuppressive molecules between the high- and low-UPR groups.** (A) Expression of *CD47* in high- and low-UPR groups. (B) Expression of *CD86* in high- and low-UPR groups. Statistical significance was determined by two-tailed *t*-test: * indicates *p* < 0.05, ** indicates *p* < 0.01, *** indicates *p* < 0.001, **** indicates *p* < 0.0001, and ns indicates not significant.

**Figure S9 Correlation between UPR scores and immune checkpoint expression.** Correlations between UPR score and immune checkpoint expression. The UPR scores were significantly positively correlated with the expression levels of *CD274*, *LGALS9*, *SIRPA*, and *PDCD1*.

**Figure S10 Correlation between stemness scores and UPR scores in pan-cancer.** Correlations between stemness scores and UPR scores in pan-cancer. The correlation coefficient is Pearson's correlation coefficient, which was used to evaluate the relationship between stemness score and UPR score in pan-cancer.

**Figure S11 Single-cell transcriptomics reveals heterogeneity in UPR activity within the TME.** (A) UMAP identified nine clusters in BRCA. (B) Markers of the nine clusters. (C) Violin plot showing the distribution of UPR scores across different cell types within the TME of BRCA patients in the same dataset.

**Figure S12 UPR activity difference in TME across different tumors.** (A) The UPR scores varied across different cells in HCC. (B) Quantification of UPR activity in the TME of HCC patients. (C) The violin plot shows hepatocellular carcinoma cells exhibit higher UPR activity compared to normal hepatocellular cells. (D) The different UPR activity in the TME of NSCLC patients. *** indicates *p* < 0.001.

**Figure S13 Characteristics of different subpopulations of macrophages.** (A) The dot plot shows the expression levels of different subpopulations of macrophages. (B-E) Enrichment analysis reveals different biological characteristics across macrophages. (F) The violin plot shows the different lipid metabolism activity across macrophages. *** indicates *p* < 0.001.

**Figure S14 Analysis of intercellular communication.** (A) The differences in cell communication frequency and intensity between the high- and low-UPR groups. (B) The difference in communication frequency and strength between tumor cells and myeloid cells in the high-UPR group compared to the low-UPR group. (C) The heatmap shows the differences in cell communication frequency and intensity between different cell clusters in the high- and low-UPR groups.

**Figure S15 UPR activity of immune cells in immunotherapy-treated patients.** The UPR activity scores of immune cells in the tumor microenvironment, calculated using AUCell, for breast cancer (A) and Merkel cell carcinoma (B). ** indicates *p* < 0.01, *** indicates *p* < 0.001, **** indicates *p* < 0.0001, and ns indicates not significant.

**Figure S16 Evaluation of the prognostic models.** (A) RT-qPCR analysis showed that TG-induced UPR significantly upregulates the expression of genes *HMT2, KDELR2,* and *SLC1A4* in MCF-7. (B) Survival curves for breast cancer patients in the TCGA cohort after adjustment for age, sex, and race using propensity score weighting. Weighted Kaplan-Meier curves are shown, and statistical significance was evaluated using the weighted log-rank test. (C-F) ROC analysis was employed to validate the effectiveness of four models in predicting the prognosis of BRCA patients within the validation cohort. * indicates *p* < 0.05, ** indicates *p* < 0.01.

**Figure S17 Kaplan-Meier curves of overall survival stratified by UPR predictors.** Kaplan-Meier curves of OS according to the UPR predictors in TCGA-ACC (*n* = 77), TCGA-CESC (*n* = 308), TCGA-BLCA (*n* = 426), TCGA-KICH (*n* = 91), TCGA-LGG (*n* = 523), TCGA-LUAD (*n* = 574), TCGA-MESO (*n* = 87), TCGA-PAAD (*n* = 183), TCGA-SARC (*n* = 264), TCGA-THCA (*n* = 571).
